# Wearables and behavioral coding show promise for measuring and predicting severe emotional outbursts in children

**DOI:** 10.3389/fdgth.2025.1641845

**Published:** 2026-01-09

**Authors:** Guido Mascia, Hannah E. Frering, Robert R. Althoff, Erieshell Coney, Diana Hume Rivera, Za’Kiya Toomer-Sanders, Christine Erdie-Lalena, Mary Dame, Laura Beth Brown, Deborah Evans, Ryan S. McGinnis, Ellen W. McGinnis

**Affiliations:** 1 Center for Remote Health Monitoring, Department of Biomedical Engineering, Wake Forest School of Medicine, Winston-Salem, NC, United States; 2 Department of Psychiatry, Larner College of Medicine, University of Vermont, Burlington, VT, United States; 3 Research Experience for Undergraduates, Wake Forest School of Medicine, Winston-Salem, NC, United States; 4 Therapeutic Day Program, Atrium Health Wake Forest Baptist, Winston-Salem, NC, United States

**Keywords:** children, electronic health records, physiology, severe emotional outbursts, wearables

## Abstract

**Introduction:**

Temper tantrums are common in early childhood. Severe emotional outbursts, however, are transdiagnostic, disruptive, and difficult to measure across settings, highlighting the need for better methods to identify and predict these components of emotion dysregulation. To address major methodological gaps, we propose a multimodal approach combining a retrospective electronic health record (EHR) analysis (Study 1) and a pilot wearable feasibility study (Study 2) to explore new ways of predicting and quantifying emotional outbursts in children enrolled in a therapeutic day program (TDP).

**Methods:**

In Study 1, we explored retrospective data collected from the EHR (historical patient data and hourly behavioral observations), trying to understand which variables might predict an outburst. In Study 2, wearable technology was employed to characterize outbursts leveraging free-living data collected during a typical day at a TDP. Moreover, we used these data to assess the future of possible outburst predictions among a clinical sample by analyzing the feasibility of such a technology.

**Results:**

An EHR analysis of 45 patients aged 4–8 years revealed that observed rough behaviors at the beginning of the day were associated with an increased likelihood of subsequent outbursts (*p* < .001), from 6% for those with zero rough behaviors to 68% for those with two or more such behaviors. Wearable sensor data demonstrated high tolerability (all four children assented each of 3–5 days of participation for 5 h of wear) and minimal data loss (<4%). Case studies of wearable-derived heart rate, heart rate variability, and skin temperature suggested that these factors might serve as promising indicators for detecting distress and outbursts.

**Discussion:**

Our results suggest that behavioral observation has the potential of predicting outbursts, and that wearable sensors are tolerable and feasible for children to wear. Overall, multiple methodologies should be studied concurrently and may be required to predict outbursts in the future.

## 1 Introduction

Displays of anger or distress and physical aggression in early childhood, described as “temper tantrums,” are common and developmentally appropriate (1). However, some children also experience impairing emotional outbursts that are defined as being “grossly out of proportion in frequency, intensity, and/or duration to the provocation” (2). These severe emotional outbursts are thought to occur in concert with atypical autonomic nervous system activity (3, 4) in response to external stressors. While severe emotional outbursts are a component of many diagnoses in childhood, they themselves can lead to impairment, comorbidity, and negative outcomes in adulthood if left untreated (5, 6). Children who experience impairing emotional outbursts and subsequent irritability (7), cause disruptions in home and school settings, and are one of the most common reasons for referral to emergency departments, inpatient hospital units, outpatient clinics, and residential treatment facilities (2). Because of the transdiagnostic, frequent, and disruptive nature of these outbursts on familial structure, school systems, and the healthcare system, there is a marked need for new preventive interventions that can support caregivers, teachers, and clinicians in managing severe emotion dysregulation in children and adolescents (8).

Effective intervention depends on anticipating outbursts and intervening on maladaptive coping strategies (2). The development of interventions for severe emotional dysregulation is challenged by the reality that outbursts are categorically difficult to predict. There are a variety of assessments and questionnaires that attempt to describe the intensity and longevity of a child's irritability and the resulting outbursts, most of which are completed by a caregiver, teacher, or clinician (9). However, these existing assessments are mostly retrospective and therefore cannot predict when an outburst may occur in time to deploy preventative intervention. Identifying antecedents to outbursts requires in-depth personalized behavioral analysis by trained personnel and assumes that antecedents can be continuously monitored reliably (10). In practice, these antecedents cannot be continuously monitored, so outbursts often feel spontaneous. When they occur, standard interventions are to remove the child from their environment, which can involve hands-on physical restraint, and the potential for physical injury to the child and those trying to help (11), along with transferring them for specialized and costly care, often through emergency departments (12).

There is scant literature on predicting outbursts across settings despite many experts in the field continuing to identify a distinct need (2, 8). In fact, it is not yet clear how to best measure these unique physiological events and their associated antecedents. Severe emotional outbursts occur infrequently in community samples, which has, to date, rendered the high effort and cost of interventionists behaviorally coding their antecedents impractical (10). As a result, it is not yet clear what behaviors might provide reliable indicators of a pending outburst. Similarly, it is not clear whether changes in a child's physiology related to their autonomic nervous system activity could serve as reliable, objective predictors of outbursts. New wearable technologies may provide a novel, relatively unobtrusive, solution for capturing objective behavioral and physiological measures (13, 14), but many of these technologies are not designed with children in mind (15), and it is not clear whether those that are, are feasible for use in children who experience severe emotional outbursts.

In this pilot work, our overarching aim is to explore potential methods for quantifying outbursts and their associated antecedents. We consider multimodal patient data collected from a mental health therapeutic day program (TDP) that treats 4–8-year-old children with severe emotional dysregulation. Data were collected in two studies: (1) a retrospective study of electronic health record (EHR) data collected in the course of standard-of-care treatment at the TDP and inclusive of caregiver interviews and observational data collected by qualified professionals (QPs); and (2) a prospective feasibility study of multimodal wearable devices that provide continuous monitoring of physiological and behavioral data from patients attending the TDP. These data were used to assess the feasibility of deploying wearable sensors in a sample of young children who experience severe emotional outbursts. Further, these data were used to identify potential markers available from these sensors, and the EHR, that predict outbursts and should be explored in future work.

## 2 Methods

### 2.1 Therapeutic Day program

The TDP is a mental health service for children aged 4–8 years referred for severe emotional dysregulation after school expulsion. Two treatment rooms of 7–8 children receive group evidence-based interventions at the TDP from 9 am to 2 pm daily for up to 9 months before being transitioned back into their public-school systems. Qualified professionals including licensed social workers, speech and language pathologists, and behavioral interventionists deliver interventions [including the Incredible Years curriculum (16)] throughout the day with daily group recreational and music therapy, as well as weekly individual occupational therapy as needed.

#### 2.1.1 Clinician engaged research

The research team engaged with the TDP team to discuss the feasibility of both the retrospective electronic health study and the wearables tolerability study, and their expertise helped inform study protocols. For instance, the TDP team recommended allowing child participants to choose stickers to put on their sensors to make the experience more enjoyable.

### 2.2 Measuring outburst events

As standard of care, QPs document every event in which a child experiences a severe emotional outburst where intervention is necessary. Unless unsafe to remove the patient from their environment, QPs bring the child into a safe, low-stimulation space. These events are called outburst events. During outburst events, QPs follow safety protocols implementing individual patient intervention plans, which may include brief physical holds and/or behavioral interventions. All outburst events and their timing and durations are documented in the EHR.

### 2.3 Study 1: Retrospective EHR study

#### 2.3.1 Procedure

The EHRs of past and present patients who attended a TDP were reviewed following postquarantine protocols, with admission dates between 1 September 2020 and 1 June 2024. Forty-five EHR records were available for review with both clinical intake data and any daily behavioral data. Research team members viewed patient files and collected relevant data using REDCap electronic data capture tools hosted at Wake Forest School of Medicine. All data entries were validated by at least two researchers.

#### 2.3.2 Data sources

##### 2.3.2.1 TDP clinical intake with patient caregiver

A clinical intake form was completed upon TDP admission via a semistructured interview administered by the TDP psychologist to the patient's caregiver. The following items were transcribed from the intake form to our dataset: gender, race, ethnicity, admission date, traumatic events experienced, medical problems experienced, mental health diagnoses, and unmet patient family needs (food, housing, transportation, health, medication, employment, financial, childcare, clothing, household, and other), Child Behavior Checklist (17) (CBCL) completed by the parent about the child at the time of admission.

##### 2.3.2.2 Data from daily TDP intervention

As part of the standard of care at the TDP, QPs tally child behaviors (i.e., “rough behaviors,” “unkind behaviors,” and “elopements from treatment rooms”) from 9 am to 10:30 am (Period 1), from 10:30 am to 12 pm (Period 2), and from 12:30 pm to 2 pm (Period 3) each day the child is present and log them into the patient's record. In this study, it was found that elopement was rare (observed only eight times across five children) and was thus left out of subsequent analyses. Unkind behaviors were highly correlated with rough behaviors (*r* = 0.73), and given the clinician input that rough behaviors were more clearly defined and demonstrated greater subjective interrater reliability, only rough behaviors were retained for analyses.

### 2.4 Study 2: Prospective wearables feasibility and data quality study

#### 2.4.1 Procedure

**Figure 1:**
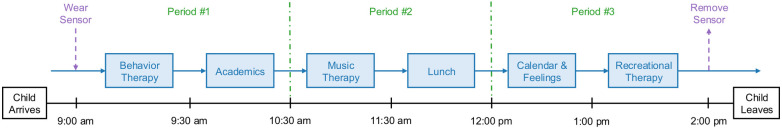
A flowchart representing a typical day at a TDP. The blue boxes represent the activities that the classroom is involved at a given time; the typical day is divided in three periods, starting approximately at 9:00 a.m., 10:30 a.m., and 12:00 p.m., respectively. The purple arrows refer to the approximate times when the sensor was applied and removed as a part of Study 2 only.

Four participants were recruited from the youth who were currently enrolled at the TDP (Table 6). QPs approached youths’ caregivers and briefly described the study, giving them an informational study flyer for caregivers to verbally assent to be contacted by the research team. The research team reached out to interested caregivers and met in-person to answer any questions and perform informed written consent. The study aimed to recruit at least four participants for 5 days each. After the consent visit with the caregiver, the participants (child patient and caregiver) were met at the beginning of their next TDP day by the research administrator. The research administrator introduced the sensor and asked for verbal assent from the child, who was then able to choose a custom sticker to be applied on the sensor. The sensor was adhered to the child's chest, and the caregiver completed a five-item survey about their child's behavior in the last 12 h. The child attended their typical TDP itinerary with the research administrator observing them to trouble-shoot sensor connectivity problems, and with an ambient noise sensor to measure each room throughout the day. All research administrator activities were recorded, and all relevant child activities were annotated. The sensor was removed prior to the child leaving the TDP each day, and the procedure was attempted for all 5 days. A schematic depiction of 1 day of recording is presented in Figure 1.

#### 2.4.2 Data sources

##### 2.4.2.1 Wearable sensor

Verbal assent to wear a flexible chest sensor (Sibel ANNE Chest, Sibel Health, Chicago, IL, USA) was obtained for each day of recording. The sensor unit consisted of an accelerometer (sampling frequency = 1,666 samples/s; range = ± 4 g), two leads for monitoring the heart electrocardiographic activity (sampling frequency = 512 samples/s), and a surface temperature-sensitive element (sampling frequency = 0.25 samples/s) (13, 18). A silicone gel adhesive was applied on the sensor, which, in turn, was attached to the participant's chest, halfway between the clavicles and the sternum, as per manufacturer recommendations. The sensor started the data collection as soon as the leads detected the first skin contact. Raw data were collected for the whole duration of the TDP day. Afterward, the sensor was removed, the adhesive disposed, and the raw data uploaded to the Sibel cloud where it was internally processed. Quantities of interest were extracted from the processed data including the participant's heart rate (HR), short-term heart rate variability via root mean square of successive differences (HRV), skin temperature (ST), and an overall measure of their activity levels, operationalized as the norm of the acceleration measured by the chest sensor, for the duration of the TDP day. These data were aggregated and resampled as needed for the visualization and analysis reported herein. Physiological (HR, HRV, and ST) and movement measures have been related to autonomic nervous system activity and may help elucidate outburst-related physiology and behavior (19, 20).

##### 2.4.2.2 Ambient noise sensor

To assess possible external sources of distress, the ambient noise level was measured using a sound level meter (R1620, REED Instruments, Wilmington, NC, USA). The recording day started following the Sibel sensor application to the participant, while the research assistant was in the same physical space (e.g., room, playground, etc.,) as the participant. The ambient noise sensor was positioned by the researchers in the same room as the participant during each activity or was as close as possible to the children when they were playing outside.

##### 2.4.2.3 Observed tolerability checklist

Tolerability was assessed via a checklist of five questions completed by an observing research team member in the treatment room for the entirety of participation (9 a.m.–2 p.m. each day). Questions included: (1) Did the child assent for the wearable to be put on? (2) Did the child take the wearable off prior to the end of the day? (3) Did the child verbally complain about the wearable sensor during wear? (4) Was the skin visibly irritated after the wearable was removed? and (5) Did the child assent to future wear of the sensor?

##### 2.4.2.4 Caregiver-report questionnaire

Caregiver questions were created with TDP administration and QP staff and asked about the participant's mood and behavior from the previous evening, night, and morning prior to TDP attendance at 9 a.m. Final questions included 6 items about how the child ate (quality/quantity), slept (quality/quantity), whether they had taken all prescribed medications, stressful events that impacted their child, and whether their child experienced an outburst of more than 10 min while not being at the TDP.

### 2.5 Data analysis

#### 2.5.1 Study 1

To compare patients who had frequent outburst events (high frequency) with those who did not have frequent outburst events (low frequency) in terms of demographics and mental health information from clinical intake, we conducted a series of independent sample *t*-tests and chi-square tests.

Generalized linear mixed models were used to identify associations between daily behavioral codes (rough, unkind, and elopement behaviors) and patient outburst events to handle longitudinal data repeated measures and to account for within-subject correlations by including random effects. The *α*-value for significance testing was set at 0.05 for hypothesis testing. One-way ANOVAs were used to compare rough behaviors across periods by outburst event timing and interpret findings from significant generalized linear mixed models with Bonferroni corrections.

#### 2.5.2 Study 2

##### 2.5.2.1 Sensor tolerability and feasibility

Chest sensor tolerability was assessed by inspecting the number of purposeful removals, verbal complaints, skin irritations, number of times the sensor fell off, and end-day child assent to future wear during the available recording days. The feasibility analysis of ambient noise was instead based on examining the percentage of missing samples across all recordings.

#### 2.5.3 Caregiver-reported data

Caregiver-reported data were analyzed by describing the frequency of the outcomes extracted from the provided questionnaire (eating, sleeping, medication, stressful events, and outbursts at home).

##### 2.5.3.1 Case study

A day where one child experienced an outburst event was chosen as a case study. The available modalities of the chest sensor were analyzed in different time periods to appreciate the physiological changes detected by each of them. In doing so, the baseline for each modality was computed as the average individual modality value during the 15 min preceding the first TDP activity (9:15–9:30 a.m.).

A subsequent analysis was performed on the available short-term HRV for the same subject for all the available recording days. The presence of possible differences between the day when the severe outburst occurred and the others was assessed via one-way ANOVAs.

## 3 Results

### 3.1 Study 1: Retrospective EHR analysis

Patient data from clinical intakes, CBCLs, daily coded behavior problems, and outburst events from 45 patients across treatment days are available in Table 1.

**Table 1:** Demographic and behavioral data extracted from the EHR.

Patient data	% (*n*) or mean (SD), range *N* = 45
Clinical intake forms (*N* = 45)	
Total treatment days	223.39 (122.63) (R: 24–735 days)
Gender (4 missing)	85% Male (35)
Age (months)	64.27 (14.80) (R: 42–95)
White	53% (24)
African American	38% (17)
Hispanic	7% (3)
Medication number (5 missing)	3.07 (2.37)
Diagnosis number (5 missing)	2.17 (.83)
ADHD	58% (26)
Anxiety disorders	44% (20)
PTSD	33% (15)
Autism spectrum/sensory disorders	11% (5)
Conduct (temper dysregulation)	71% (32)
History of traumatic experiences	
Physical abuse	36% (16)
Sexual abuse	11% (5)
Witnessed family member violence	47% (21)
Family member with substance use	36% (16)
Family member with mental illness	80% (36)
Other traumatic events	27% (12)
Traumatic experiences sum (R: 0–6)	2.71 (1.92)
History of medical problems	
NICU/ED/surgery required	27% (12)
In utero drug exposure	20% (9)
Gross motor or speech delay	11% (5)
Household unmet needs:	
0	51% (23)
1	22% (10)
2+	27% (12)
CBCL parent report (5 missing)	
Internalizing T score	64.20 (9.33)
Externalizing T score	72.10 (8.79)
Behavioral data (*N* = 697 days)	
Period 1 rough behaviors (*n* = 697)	0.36 (1.4) R: 0–13
Period 2 rough behaviors (*n* = 690)	0.38 (1.5) R: 0–12
Period 3 rough behaviors (*n* = 688)	0.35 (1.3) R: 0–14
Outburst events (*N* = 1,762 days)	
Outburst events per child	26.48 (34.99) (R: 0–136)
Outburst events of total treatment days	18.45% (15%) (R: 0%–58%)
Percent of patients with ratio >15%	26% (11/45)
Outburst duration (min)	17.41 (15.44) (R: 4–60 m)
Outburst event timing	
Days with no outburst	32% (570)
Outburst any period	68% (1,192)
Outburst in period 1	22% (387)
Outburst in period 2	30% (526)
Outburst in period 3	16% (279)

SD, standard deviation; R, range; ADHD, attention-deficit/hyperactivity disorder; PTSD, posttraumatic stress disorder; NICU, neonatal intensive care unit; ED, emergency department; CBCL, child behavior checklist.

No variables from the clinical intake were associated with high vs. low frequency of outburst event incidence (> or <15% outburst days to total treatment days) using *t*-tests or chi-square tests (see Table 2).

**Table 2:** Comparison of patients by outburst event frequency (low vs. high frequency) in terms of demographics and mental health information from clinical intake.

Patient data	Low frequency (*n* = 34) <15% Outburst days	High frequency (*n* = 11) >15% Outburst days	*P*-value
Age (months)	65.00 (15.84)	59.55 (11.81)	.228
White	53.1% (17)	63.6% (7)	.545
Medication number	2.81 (2.43)	3.55 (2.38)	.379
Diagnosis number	2.28 (.85)	2.00 (0.77)	.340
ADHD	56.3% (18)	63.6% (7)	.668
Anxiety	50.0% (16)	36.4% (4)	.434
PTSD	34.4% (11)	36.4% (4)	.905
Conduct	71.9% (23)	63.6% (7)	.608
Internalizing T score	65.41 (9.26)	60.70 (9.58)	.177
Externalizing T score	72.82 (9.06)	70.50 (8.50)	.482
Transportation need	28.10% (9)	27.3% (3)	.957

Rough behaviors within each TDP period were compared across the following outburst event groups: no outburst events, outburst event in Period 1, 2, or 3, as presented in Table 3. For days with no outburst events, there were no periods with elevated (mean >1) rough behaviors. For days when an outburst event occurred in Period 1 or Period 2, there were elevated rough behaviors across Periods 1, 2, and 3 (*p* < .001). Therefore, days with outburst events in Periods 1 or 2 were analyzed together in subsequent linear mixed models, as elevated rough behaviors across the day were similar for both. For days when an outburst event occurred in Period 3, Period 3 was the only period with elevated rough behaviors.

**Table 3:** Comparison of rough behaviors by period to outburst event timing.

Outburst event timing	Rough behaviors		
	Period 1	Period 2	Period 3
None	0.11^a^ (0.55)	0.08^a^ (0.45)	0.11^a^ (0.79)
Period 1 (387)	**3.11^b^ (3.03)**	**2.49^b^ (3.55)**	**1.63^b^ (2.78)**
Period 2 (526)	**1.75^b^ (3.02)**	**2.03^b^ (3.00)**	**1.21^b^ (2.15)**
Period 3 (279)	0.17^a^ (0.50)	0.36^a^ (2.00)	**1.39^b^ (1.96)**

Bonferroni-adjusted ANOVAs show different letters all <0.001.

Statistically significant results are in bold.

In linear mixed models (see Table 4), rough behaviors from Periods 1 and 2 were both associated with having an outburst event in Periods 1 and 2. Rough behaviors from Period 3 were associated with having an outburst event in Period 3. No clinical intake data were significantly associated with having an outburst event in any period.

**Table 4:** Association of period rough behaviors with outburst events in Periods 1, 2, and 3 using generalized linear mixed models.

LMM predict outburst 1 + 2 vs. none or 3	Est (std error)	*p*	OR
Period 1 rough behaviors	**0.113 (0.009)**	**<0**.**001**	**1**.**12**
Period 2 rough behaviors	**0.099 (0.009)**	**<0**.**001**	**1**.**10**
LMM predict outburst 3 vs. none			
Period 1 rough behaviors	−0.012 (0.007)	0.083	
Period 2 rough behaviors	−0.002 (0.007)	0.758	
Period 3 rough behaviors	**0.036 (0.007)**	**<0**.**001**	**1**.**04**

Statistically significant results are in bold.

In *post-hoc* analyses of significant findings from generalized linear mixed models, rough behaviors in Period 1 were associated with the likelihood of having an outburst event in Period 1 or 2 (*p* < .001), which increase from 6% for those with zero Period 1 rough behaviors, 53% for those with one Period 1 rough behavior, and 68% for those with two or more Period 1 rough behaviors. Only rough behaviors in Period 3 were associated with the likelihood of having an Outburst event in Period 3 (*p* < .001), which increase from 3% for those with zero Period 3 rough behaviors, 32% for those with one Period 3 rough behavior, and 21% for those with two or more Period 3 rough behaviors (Table 5).

**Table 5:** *Post-hoc* analyses of outburst likelihood by rough behavior increase in periods 1–3.

Period 1 rough behaviors	Outburst 1 + 2	Outburst 3
0	6% (36)	5.3% (32)
1	53% (24)	6.7% (3)
2+	68% (32)	2.1% (1)
*P*-value	**<0.001**	0.574
Period 3 rough behaviors	Outburst 1 + 2	Outburst 3
0		3% (18)
1		32% (8)
2+		21.3% (10)
*P*-value		**<0.001**

Statistically significant results are in bold.

### 3.2 Study 2: Prospective wearables feasibility and data quality study

#### 3.2.1 Participants

Four child participants were recruited into the study (see Table 6 for demographic data). Two participants stopped participation after three days for reasons unrelated to the research study (no longer enrolled at the TDP or weather-related cancelations at the TDP; TDP absences).

**Table 6:** Participant demographics from study 2.

ID	Age	Sex	Days	Outbursts
S1	7	M	3	3
S2	5	M	5	1
S3	7	F	5	0
S4	5	M	3	1

#### 3.2.2 Sensor tolerability

*Assent*: All four children assented to the wearable each day of participation. *Purposeful removal*: One of the four children removed the wearable one time 10 min into an outburst event. *Verbal complaints*: Two children complained about the sensor “itching” 1–3 times with no requested intervention. One child complained about itching 13 times, requesting brief sensor removal periods (4% of planned wear time), which QPs note may have been associated with excitement to interact with research personnel. *Skin irritation*: One child had visible skin irritation once after removal. *Assent to future wear:* At the end of each day, all children assented to next day wear. *Sensor/adhesive feasibility note:* Sensors fell off children 11 times, seemingly because of summer heat when playing outside (°C average ± SD: 28.0 ± 3.5; °F average ± SD: 82.4 ± 6.3). During these times, children typically notified the researcher. In one instance, a sensor fell off a child 10 min into an outburst event and was not put back on until after the outburst event had finished.

#### 3.2.3 Ambient noise feasibility

Two out of 16 days of recordings were unavailable due to sensor failure. Average data missingness was approximately 1% (70 s over 4:15 h, on average). *Feasibility note*: Research administrators noted that colocating off-body sensors to be near child participants was burdensome.

#### 3.2.4 Caregiver-report data

Three of the four caregivers completed daily questionnaires. *Eating*: The average response across three participants (over 13 days) was “They ate some (dinner/breakfast), but it was not good quality/or they did not eat enough food.” *Sleeping*: The average response was “They did not sleep enough and/or it was not good quality sleep.” *Medication*: All children were reported as having taken their prescribed medication each day. *Stressful Events*: One child experienced one minor stressful event on one day; otherwise, no stressful events were reported for any other children. *Outbursts at home*: One child experienced an outburst lasting more than 10 min on three of their five participation days (see Figure 3).

#### 3.2.5 Case study

**Figure 2:**
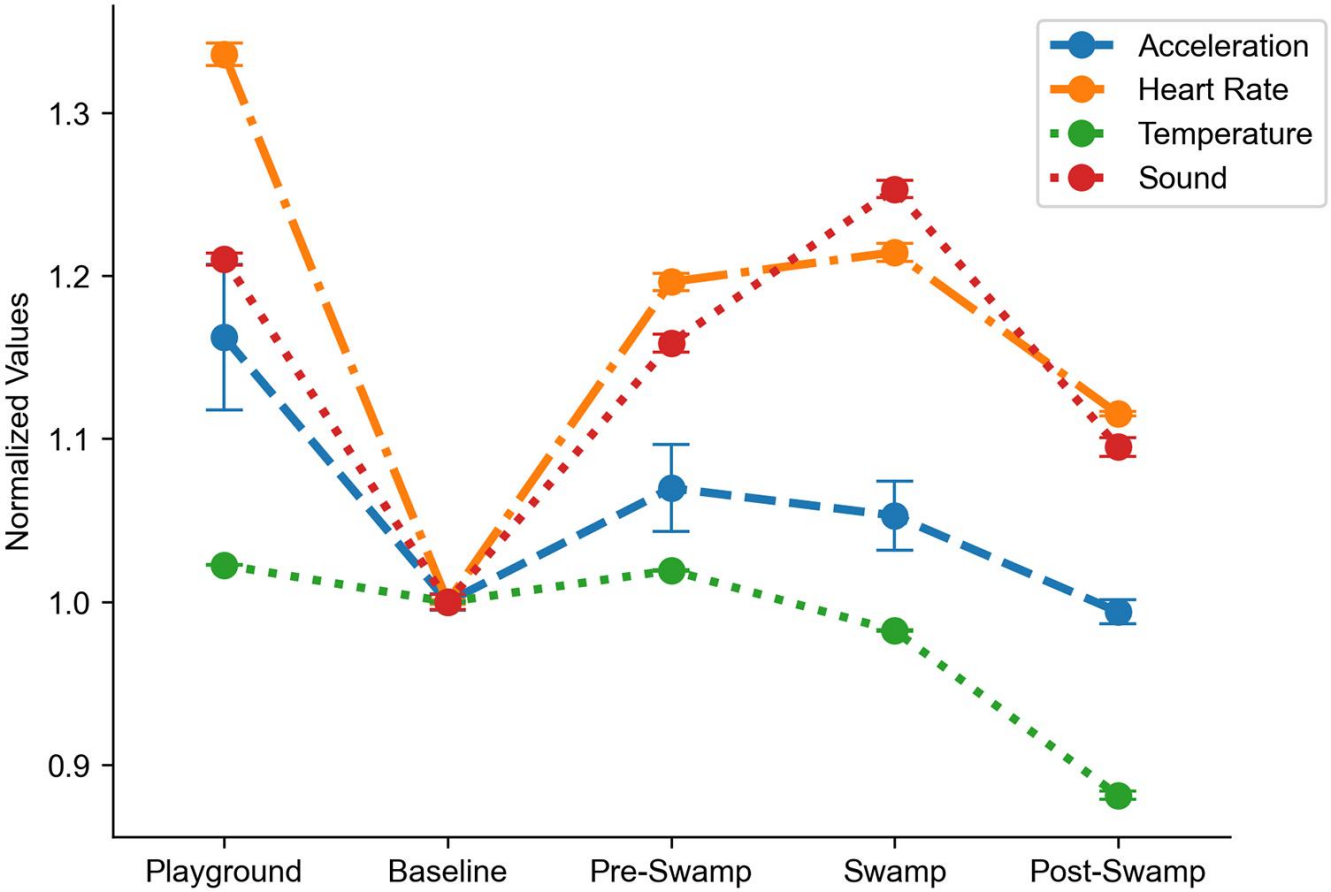
A point plot representing the mean values along with the corresponding standard error of the squared acceleration norm (blue), heart rate (orange), temperature (green), and sound (red) for the selected periods of the day. Values have been normalized based on the ones at baseline, identified in the 15 min prior to the beginning of the behavior therapy (9:15 a.m. to 9:30: a.m.). The SWAMP and post-SWAMP values refer to only those instants when the sensor was attached. SWAMP stands for *Sit, Wait, and Make a Plan*.

**Figure 3:**
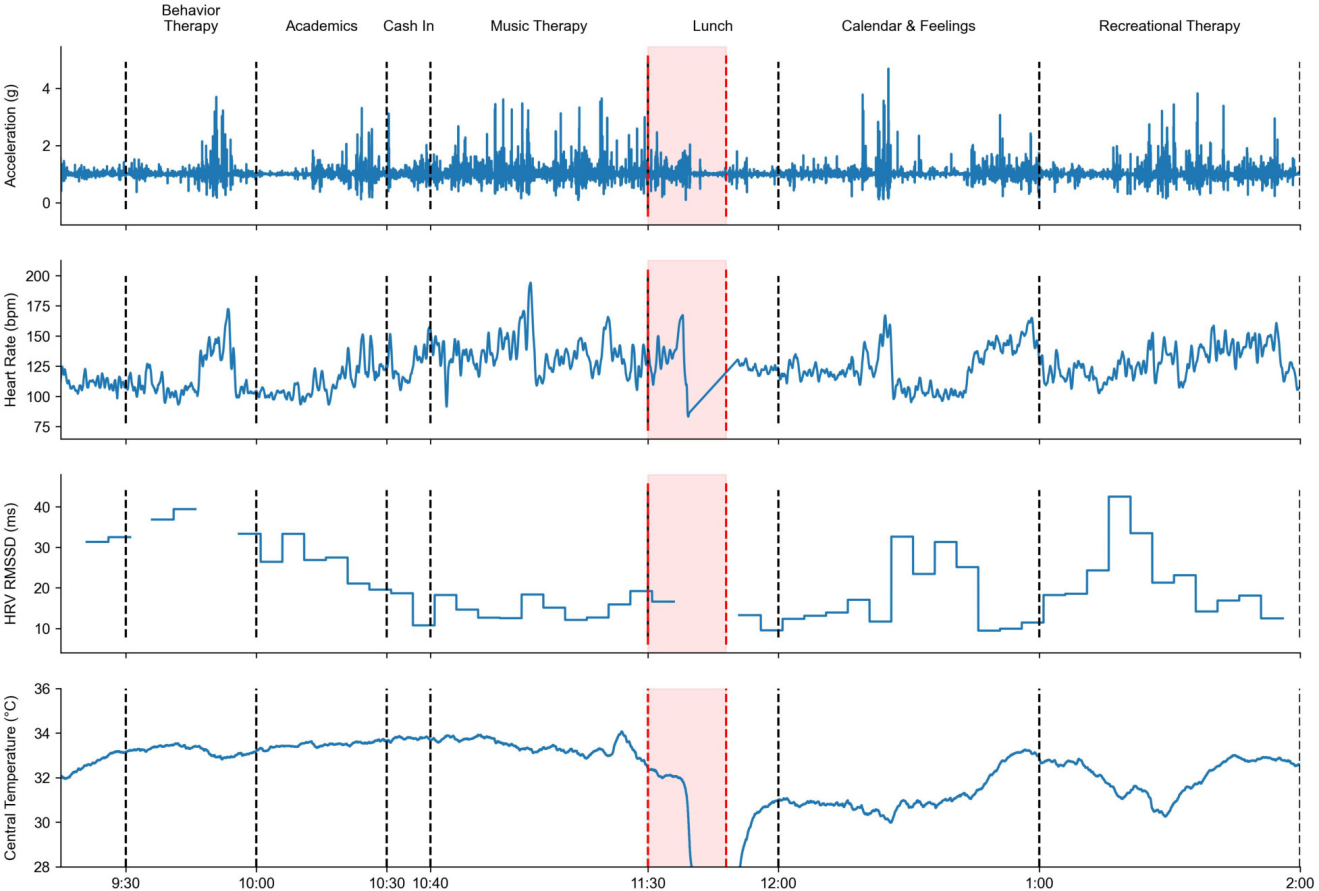
Sensor modalities for an entire TDP day. Each sample corresponds to 1 s of recordings. The dashed black lines indicate a transition from one activity to the other. The red dashed lines indicate SWAMP occurrence. It is possible to identify the sensor detaching from the participant halfway through the SWAMP (CT dropped, HRV missing values, and HR abnormal pattern). No insights can be drawn from the accelerometer alone.

See Figure 2 for sensor modalities within the selected period of the day (using 10-min intervals) for comparison of playing, sitting, time just prior to an outburst, during an outburst, and time immediately post outburst. Data recorded during an example TDP day inclusive of an outburst event are presented in Figure 3.

**Figure 4:**
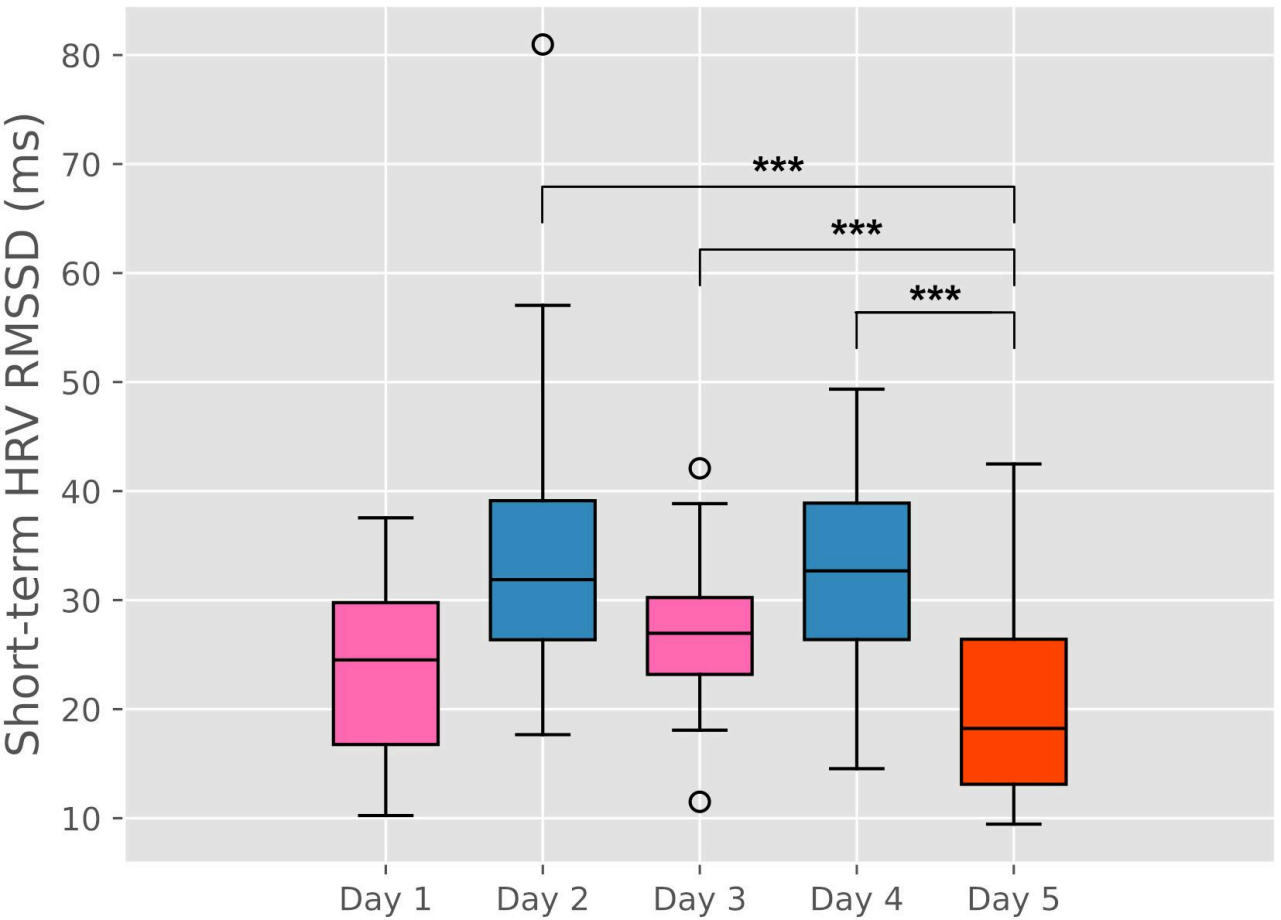
Aggregated values for short-term HRV for each day at the TDP. The red box corresponds to the day when the participant experienced an outburst that brought to a SWAMP. Missing values have been excluded from such analysis. ***: *p* = 0 in the independent sample *t*-test.

See Figure 4 for daily aggregate HRV by day. Interestingly, although Day 5 was the only day inclusive of an outburst (duration of 20 min at Period 3), Days 1, 3, and 5 were indicated by the caregiver as days when the child experienced an outburst lasting more than 10 min the last evening before bed or morning prior to attending the TDP at 9 a.m. The caregiver reported that there were no differences across days with regard to the following: the child eating breakfast; (all “They did not eat enough, or it was not good quality food”); sleep (all “They did not sleep enough and/or it was not good quality sleep.”); medication (all “My child took their prescribed medication for today”); or stressful events (all “none”).

## 4 Discussion

We conducted two pilot studies to examine the utility of multiple data sources in the prediction of severe emotional outbursts by children in a therapeutic day program. First, we conducted a retrospective EHR analysis of 45 child patient records across an average of 7 months to assess whether documented lifetime and daily coded behaviors predict outbursts. We also conducted a prospective human subject study of four child patients across 5 days to test the feasibility of studying on- and off-body sensor monitoring in this population, and the quality of the resulting data with the intention of informing future study methodology on outburst prediction.

Our retrospective EHR analysis suggests that coded child behaviors can predict severe child outburst forecasting within 1–2 h. Interestingly, intake factors such as diagnoses, medications, and social determinants of health did not predict outburst frequency while in the program, potentially because of the selection bias of the sample being referred for severe emotional outbursts. QPs taking time and effort in the moment to code rough behaviors appears to be advantageous for understanding the antecedents of outbursts. At the TDP, engagement in just two rough behaviors increased the likelihood of an outburst in the next 3 h by 10-fold. Immediate behavioral coding appears to be a worthwhile personnel effort for outburst prevention, and yet the feasibility of obtaining this expertise outside contexts like the TDP is low in terms of availability of expert personnel, time, and cost. Therefore, it is valuable to pursue alternative indicators of continuous child behavior that do not require such efforts, with one such indicator being wearable sensors.

Our prospective sensor study suggests that on-body sensors are feasible to use and tolerable for children to wear. Promising feasibility is consistent with one other pilot study using a wrist-worn wearable sensor on 10 young children at high risk for emotional outbursts (21). Overall, child patients were agreeable, and furthermore, they were excited to wear sensors, although we recommended protocols for trouble-shooting sensors falling off because of sweat or heat and/or making adjustments if they felt itchy. Off-body noise sensors were somewhat burdensome for research administrators to move across the child's daily activities; alternative on-body possibilities for noise detection may be merited (22). Data from sensors suggest that outbursts may not be as obvious in behavioral and physiological data as previously assumed, such that without tagging with EHR data, a researcher would not be certain when outbursts occurred using sensor data only. It is unknown whether this phenomenon may be due to this sample (each child having multiple mental health diagnoses and prescribed psychotropic medications), which have been associated with blunted physiology during times of frustration compared with typically developing children (23). However, an increasing heart rate in the 10 min prior to outbursts and lowered heart rate variability on days with outbursts (before and during TDP programming) may be promising indicators of outburst prediction in future work.

Multiple methodologies are likely required for understanding severe outburst detection and prediction: QP hourly behavior codes from the EHR, program itinerary tagging, caregiver reports of outbursts outside the program, and sensor data each provide important context and should all be incorporated together in a future study methodology. Having said this, it could also be possible to passively detect rough behaviors and elements of the program itinerary directly from wearable sensor data, thus streamlining collection. Integrating these data sources can support the development of just-in-time mobile health interventions [e.g., (24–26)] that help prevent severe emotional outbursts and keep children in environments where they can learn and thrive.

There are some limitations of the studies and results described here. First, the analysis considered limited sample sizes for both the EHR and the wearable feasibility studies (*N* = 45 and *N* = 4, respectively). Sensor recordings were further limited by the short time span (3–5 days). Participants from only within the TDP were considered, which may limit the generalizability of conclusions to other contexts or populations. Similarly, the participants had a variety of psychiatric diagnoses with prescribed psychotropic medications, whose effects may complicate our interpretation of the physiological response during outbursts. There were also some limitations to data completeness and quality. One caregiver did not complete any of the daily questionnaires. Although these incidents did not occur frequently, technical failures such as sensor delamination did occur, with one participant experiencing delamination during an outburst. This limited our capacity to derive insights from their complete physiological response.

Future studies would benefit from the implementation of additional sensing modalities that could track physiological signals that may also be related to precursors of emotional outbursts including electrodermal activity, breath rate, electroencephalography, and ambient noise. Similarly, the presence of a control group composed of children who do not experience severe emotional dysregulation could improve our understanding of behavioral patterns that may lead to outbursts. In addition, future studies could include QP and caregiver on-body sensors to understand physiological synchrony before, during, and after severe child outbursts. Finally, future research should consider the ethical implications [see (27, 28)] of long-term continuous monitoring through wearables on children with behavioral difficulties. For instance, if wearables were used in contexts outside therapy sessions, they may stigmatize children wearing them or reduce autonomy and trust, and thus cost–benefit analyses should be thought through prior to implementation.

## Data Availability

The datasets presented in this article are not readily available because they contain sensitive personal information derived from Electronic Health Records and wearable sensors. Because of institutional and legal restrictions related to privacy protection, the data cannot be shared publicly or upon request. Requests to access the datasets should be directed to guido.mascia@advocatehealth.org.
